# Supplementary material for: Low-pass filtering revisited: ramped phase randomization for cryo-EM map comparison and data augmentation
Source: bioRxiv. 2026 Nov 1:2026.10.30.763149. Preprint. [Version 1] doi: 10.64898/2026.10.30.763149 (PMC13734142; doi:10.64898/2026.10.30.763149)
Supplement: Supplementary file 1 [file NIHPP2026.10.30.763149v1-supplement-1.pdf]

bioRxiv preprint doi: https://doi.org/10.64898/2026.10.30.763149; this version posted November 1, 2026. The copyright holder for this preprint (which was not certified by peer review) is the author/funder, who has granted bioRxiv a license to display the preprint in perpetuity. It is made available under a CC-BY 4.0 International license.

Supplementary information

S1. Coherence of wrapped-normal offsets and expected FSC between maps

For offsets drawn from a zero-mean normal distribution with standard deviation σ(s), the coherence of equation (3) is

$$c(s) = \frac{1}{\sqrt{2\pi}\,\sigma(s)} \int_{-\infty}^{\infty} \exp\left[-\frac{\theta^2}{2\sigma^2(s)}\right] \cos\theta \, d\theta = e^{-\sigma^2(s)/2}. \quad (S1)$$

The integral is equivalent to the Fourier transform of a Gaussian, which is again a Gaussian. Wrapping the offsets modulo 2π does not change cos δ. The result also holds for wrapped-normal offsets. Inverting the result gives the third relation in equation (6).

Expected FSC between independent maps. Let X and Y be two maps of the same structure S, with errors that are uncorrelated with S and with each other. In a shell with signal power P_S and error powers N_X and N_Y, the cross terms vanish in expectation. For shells with many coefficients, the FSC of each map with the ideal map is therefore $a_X = \sqrt{P_S/(P_S + N_X)}$, and the FSC between the two maps is

$$\mathbb{E}[\mathrm{FSC}_{X,Y}] = \frac{P_S}{\sqrt{(P_S + N_X)(P_S + N_Y)}} = a_X a_Y,$$

which is equation (8); it holds for any positive scaling of the signal within a shell. For half maps, $N_X = N_Y$, so $\rho_{1/2} = \mathrm{SSNR}_{1/2}/1 + \mathrm{SSNR}_{1/2} = a_{1/2}^2$. Averaging the two half maps halves the error power, so the full map has twice the SSNR and envelope $a^2 = 2\rho_{1/2}/(1 + \rho_{1/2})$. Equation (5) sets the full-map SSNR to 1/3 at $1/R$, which corresponds to a half-map SSNR of 1/6 and a half-map FSC of 1/7 ≈ 0.143. For a filtered parent H', phase offsets independent of the parent multiply its correlation with the ideal map by c, so $a_{H'} = c a_H$, whereas its expected FSC with the parent is c (equation 4). With c from equation (7), $a_{H'} = a_T$, and the expected FSC of H' with an independent map at the target resolution is $c a_H a_T = a_T^2$.

Simulation. Figure S1 checks these relations numerically, with the registered T20S parent map (EMD-6287; 256^3 grid, 1.288 Å voxels), standardized to zero mean and unit variance, as the ideal map. To link the half-map and full-map criteria, two copies of the ideal map were filtered with equation (2), each with coherence $c = \sqrt{u_R/2 + u_R}$ for $R = 8$ Å, where u_R is the full-map SSNR of equation (5), and then averaged (Figure S1A). The copies behave as half maps: their

bioRxiv preprint doi: https://doi.org/10.64898/2026.10.30.763149; this version posted November 1, 2026. The copyright holder for this preprint (which was not certified by peer review) is the author/funder, who has granted bioRxiv a license to display the preprint in perpetuity. It is made available under a CC-BY 4.0 International license.

FSC crossed 0.143 at 7.98 Å, each copy correlated with the ideal map at √(1/7) ≈ 0.378 at 8.00 Å, and their average correlated with the ideal map at 0.5 at 8.00 Å (Figure S1B). For maps of different quality (Figure S1C), half maps at resolution were simulated by adding Gaussian noise with shell power to the ideal map, so that their average follows equation (5). A full map at Å, chosen close to the target so that the predictions separate, was filtered to Å with equation (7) and compared with the parent, the ideal map and an independent full map at . Averaged over 20 draws, all simulated FSCs agreed with the predictions to within 0.006 up to 1.5/ . Averaging realizations removes the phase randomization (Figure S1D, E). Because the offset factor has mean (equation 6), the mean of independent realizations converges to the deterministic amplitude filter , which keeps the phases of the parent. Its expected FSC with the parent is , which rises from for a single map (equation 7) toward 1. For the ideal map filtered to Å, the means of 1, 4, 16 and 64 realizations agreed with this expression to within 0.009 up to 1.5/ over five draws.

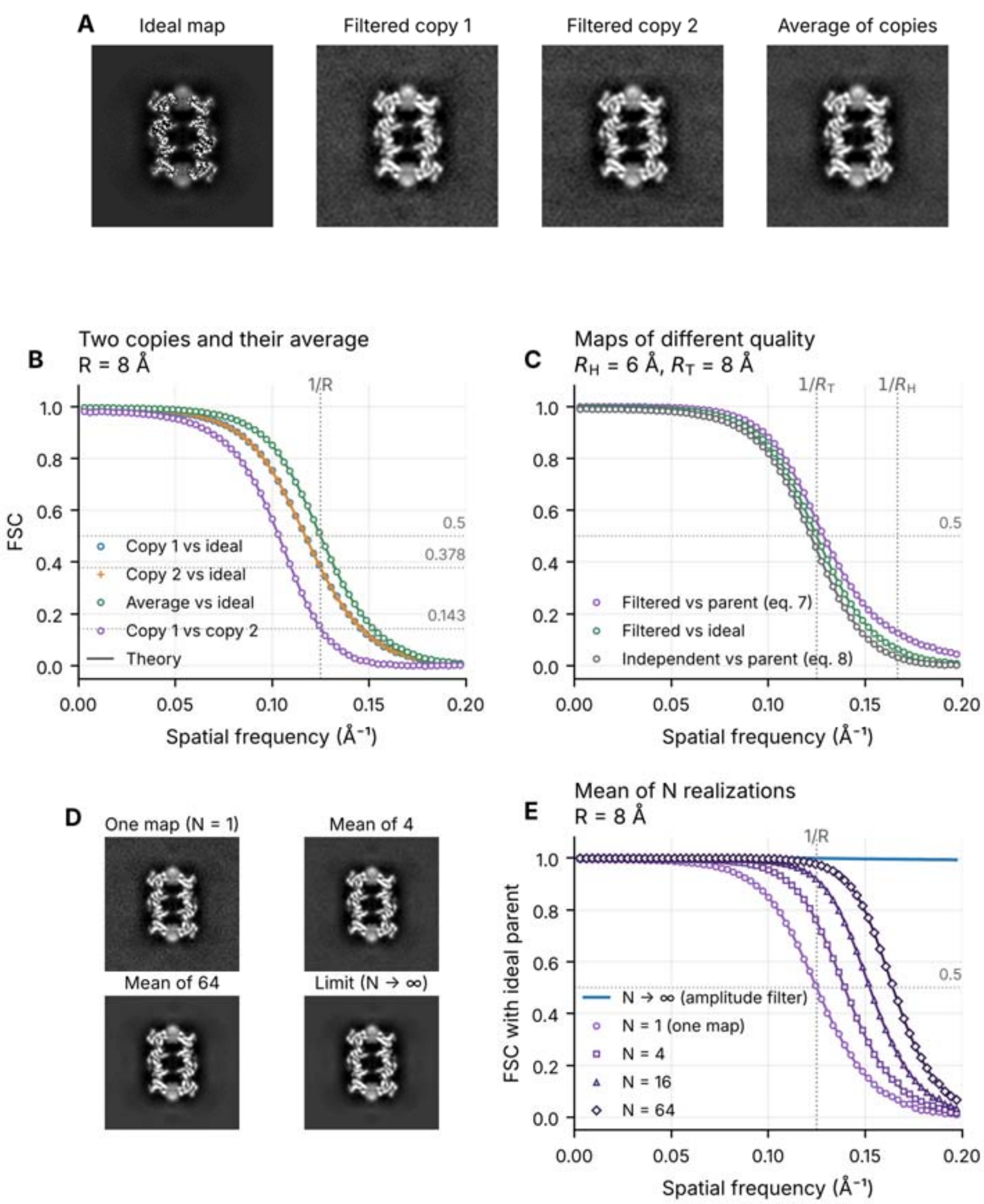

Figure S1. Expected FSCs in simulation. (A) Central sections of the ideal map (T20S parent, EMD-6287), two ramped-phase copies filtered to behave as half maps at Å, and their average. (B) FSC of each copy and of their average with the ideal map, and FSC between the

bioRxiv preprint doi: https://doi.org/10.64898/2026.10.30.763149; this version posted November 1, 2026. The copyright holder for this preprint (which was not certified by peer review) is the author/funder, who has granted bioRxiv a license to display the preprint in perpetuity. It is made available under a CC-BY 4.0 International license.

two copies. At $1/R$, the copies agree with each other at 0.143, while their average agrees with the ideal map at 0.5. (C) Maps of different quality: FSC of a filtered parent ($R_H = 6$ Å, filtered to $R_T = 8$ Å) with the parent (a_T/a_H, equation 7) and with the ideal map (a_T), and FSC of an independent full map at R_T with the parent ($a_H a_T$, equation 8). (D) Central sections of a single ramped-phase map (ideal map filtered to $R = 8$ Å), of the means of 4 and 64 independent realizations, and of their limit, the amplitude filter cT. (E) FSC of the mean of N realizations with the ideal map, which rises from c (equation 7) toward 1 as N grows. Markers show means over 20 draws (five in E), shading ±1 SD and lines the predictions. All FSCs are unmasked.

S2. Expected FSC between a filtered map and an independent map

Phase randomization multiplies each Fourier coefficient of the parent H by $e^{i\delta}$ and leaves its magnitude unchanged. The offsets δ are drawn independently of any other map L, and the expected value of $e^{i\delta}$ is the coherence c (equation 6). The expected FSC between the filtered parent H' and L is therefore $\mathbb{E}[\rho_{H',L}] = c\rho_{H,L}$, where $\rho_{H,L}$ is the FSC of the unfiltered parent with L. An amplitude response that is constant within each shell cancels in the FSC, so the prediction also holds after amplitude matching.

S3. Phase perturbation as a training augmentation

Section 3.3 compares phase augmentation with no augmentation (Figures 4, 5 and S2). This section gives the protocol for that comparison (Table S1) and summarizes additional experiments that compared phase augmentation with alternative augmentations, including geometric augmentation (Figure S3). All experiments use the offset profile of Section 5.4 with unit amplitude response, without registration or amplitude matching, and preserve conjugate symmetry and the DC term. Within each seed, the compared methods share initial weights, data order and augmentation masks. Models are evaluated at the final checkpoint without checkpoint selection, and test inputs are not augmented.

Supervised experiments for Figures 4 and 5. Table S1 lists the datasets, models and training settings.

Table S1. Datasets, models and training settings for the supervised experiments in Figures 4, 5, S2 and S3.

Setting	CryoBench Ribosembly	UCI Synthetic Control	Noisy MNIST
Data	Deposited particles of 16 Ribosembly states (Jeon et al., 2024; Qin et al., 2023) with random views, CTF and nominal SNR 0.01; 112 × 112 crops at 3 Å per pixel, backprojected onto a 144^3 grid with the supplied poses (Section 5.4)	600 traces of length 60 in six classes (Alcock, 1999); no added noise	Digits (LeCun *et al.*, 1998) low-pass limited to 0.16 cycles per pixel, plus white Gaussian noise with the rms of the centred signal (0 dB)
Seeds	9101201–9101205 (paired training seeds on fixed data)	301–325	711–735

bioRxiv preprint doi: https://doi.org/10.64898/2026.10.30.763149; this version posted November 1, 2026. The copyright holder for this preprint (which was not certified by peer review) is the author/funder, who has granted bioRxiv a license to display the preprint in perpetuity. It is made available under a CC-BY 4.0 International license.

Training set	16, 32, 64, 128 or 256 particles per state, nested, from a pool of 1,280 per state	60 labelled traces (10 per class); 12–240 in Figure S2	500 labelled images (50 per digit); 100–5,000 in Figure S2
Validation set	64 particles per state, not used for model selection	60 fixed traces	2,000 withheld training images, one fixed noise realization
Test set	256 particles per state (4,096), one fixed set	300 fixed traces	All 10,000 test digits, two fixed noise views
Classifier	2,985,984 (144^3 backprojection) → 32 → 2–16 → 16	60 → 32 → 8 → 6; latent 2–64 in Figure S2	784 → 64 → 4 → 10; latent 2–64 in Figure S2
Optimization	Adam, learning rate 0.001 (first-layer weights 1.37 × 10□□), batch size 64, 3,072 updates	Adam, learning rate 0.003, 2,000 full-batch updates	Adam, learning rate 0.001, batch size 100, 3,072 updates
Ramp start frequency	U(0.036, 0.044) cycles per pixel	U(0.9/28, 1.1/28) cycles per sample	U(0.144, 0.176) cycles per pixel
Ramp end frequency	0.20 cycles per pixel	0.25 cycles per sample	0.28 cycles per pixel

Example and embedding panels. Figure 5 shows seed 303 for UCI, chosen to illustrate the effect, and seed 711 for MNIST. Panels A and E show actual training inputs with one illustrative phase draw each. The UCI PCA is fitted on the training features and applied to the 300 test traces. For MNIST, t-SNE (van der Maaten & Hinton, 2008) embeds 1,500 randomly selected test digits from the first noise view, with perplexity 40 and a fixed initialization seed. Projections use separate coordinates and are illustrations only; the conclusions rest on test accuracy across seeds.

Cryo-EM particle classification (Figure 4). Table S1 lists the settings. We trained five paired seeds at every training-set size (16–256 particles per state) and latent dimension (2, 4, 8 and 16), giving 200 models, all tested on the same 4,096 particles. For the ARI, latent features were standardized with training statistics, and k-means with 16 clusters was fitted to the training particles, with each test particle assigned to the nearest centre. The t-SNE panels show seed 9101201 and embed all 4,096 test particles (perplexity 40, 1,500 iterations, fixed initialization seed).

Training-size and latent-dimension sweeps (Figure S2). The sweeps used the classifiers, optimizers and ramp settings of Table S1, with latent dimensions of 2, 4, 8, 16, 32 and 64 and new seeds (1301–1325 for UCI and 2301–2325 for MNIST). UCI training sets contained 12, 30, 60, 120, 180 or 240 traces. For each seed, the 600 traces were partitioned anew into 300 test traces, 60 validation traces and a 240-trace training pool, balanced across classes. MNIST training sets contained 100, 250, 500, 1,000, 2,500 or 5,000 images, trained in minibatches of at most 100 images and tested on the 10,000 test digits with the two noise views used for Figure 5. Within each seed, smaller training sets were nested subsets of the largest set, and input normalization used training-subset statistics only.

Phase augmentation increased mean test accuracy and reduced the mean train–test gap in all 36 settings for each dataset, including the 64-dimensional settings not shown in Figure S2.

bioRxiv preprint doi: https://doi.org/10.64898/2026.10.30.763149; this version posted November 1, 2026. The copyright holder for this preprint (which was not certified by peer review) is the author/funder, who has granted bioRxiv a license to display the preprint in perpetuity. It is made available under a CC-BY 4.0 International license.

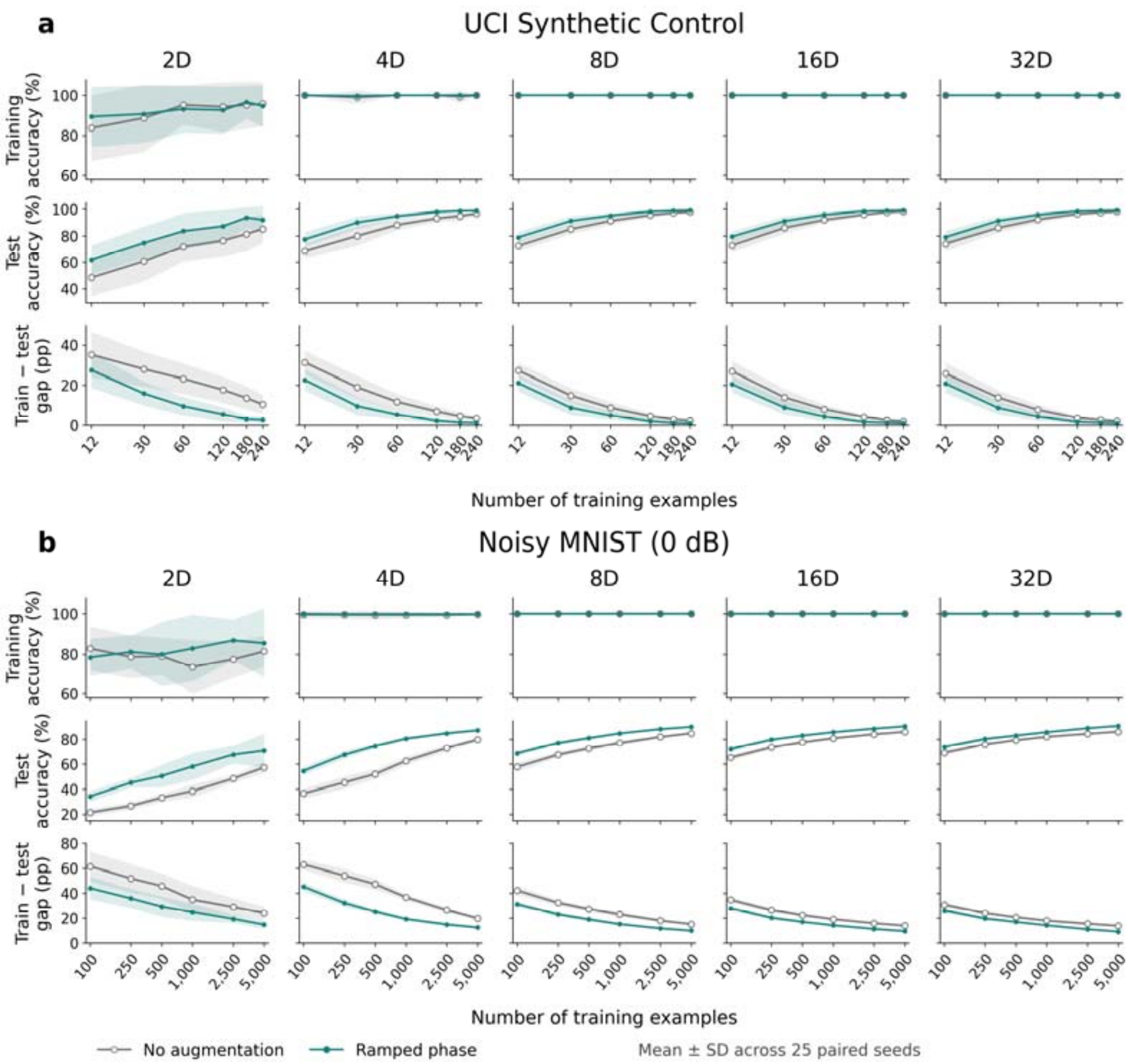

Figure S2. Phase augmentation across training-set sizes and latent dimensions for (a) UCI Synthetic Control and (b) noisy MNIST. Columns show latent dimensions of 2–32, and rows show training accuracy, test accuracy and the train–test gap against the number of training examples on a logarithmic axis. Grey open circles show no augmentation; teal filled circles show ramped phase augmentation. Lines and shading show mean ± 1 SD over 25 paired

Training accuracy, test accuracy and the gap are shown together, because a smaller gap can reflect either better generalization or poorer fitting of the training data. At latent dimensions of 8–32, both conditions fitted every UCI training set perfectly and every MNIST training set to at least 99.9%. In these settings, mean test accuracy still improved by 0.99–6.43 percentage points for UCI and 3.74–11.03 percentage points for MNIST, consistent with reduced overfitting and more robust generalization. For UCI, gains were largest for the smallest training sets and fell to about one percentage point at 240 traces with 8–32 latent dimensions, where test accuracy without augmentation was already near 98%. For MNIST, the largest gains occurred with two and four latent dimensions, and the setting of Figure 5 (500 images, four dimensions) gave the largest gain in the grid (22.5 percentage points with the sweep seeds). With two latent dimensions, models sometimes underfit, and gap changes there reflect training fit as well as generalization.

Comparison with geometric augmentation. Geometric transformations have long been used to augment handwritten-digit training data (LeCun *et al.*, 1998). To test whether phase augmentation adds to geometric augmentation, the MNIST sweep also used rotation within ±15° and translation within ±2 pixels, alone and after phase augmentation, with matched seeds,

bioRxiv preprint doi: https://doi.org/10.64898/2026.10.30.763149; this version posted November 1, 2026. The copyright holder for this preprint (which was not certified by peer review) is the author/funder, who has granted bioRxiv a license to display the preprint in perpetuity. It is made available under a CC-BY 4.0 International license.

training subsets and initializations (Figure S3). The transformations were applied to the noisy training images. Across the 30 displayed settings, phase augmentation alone raised test

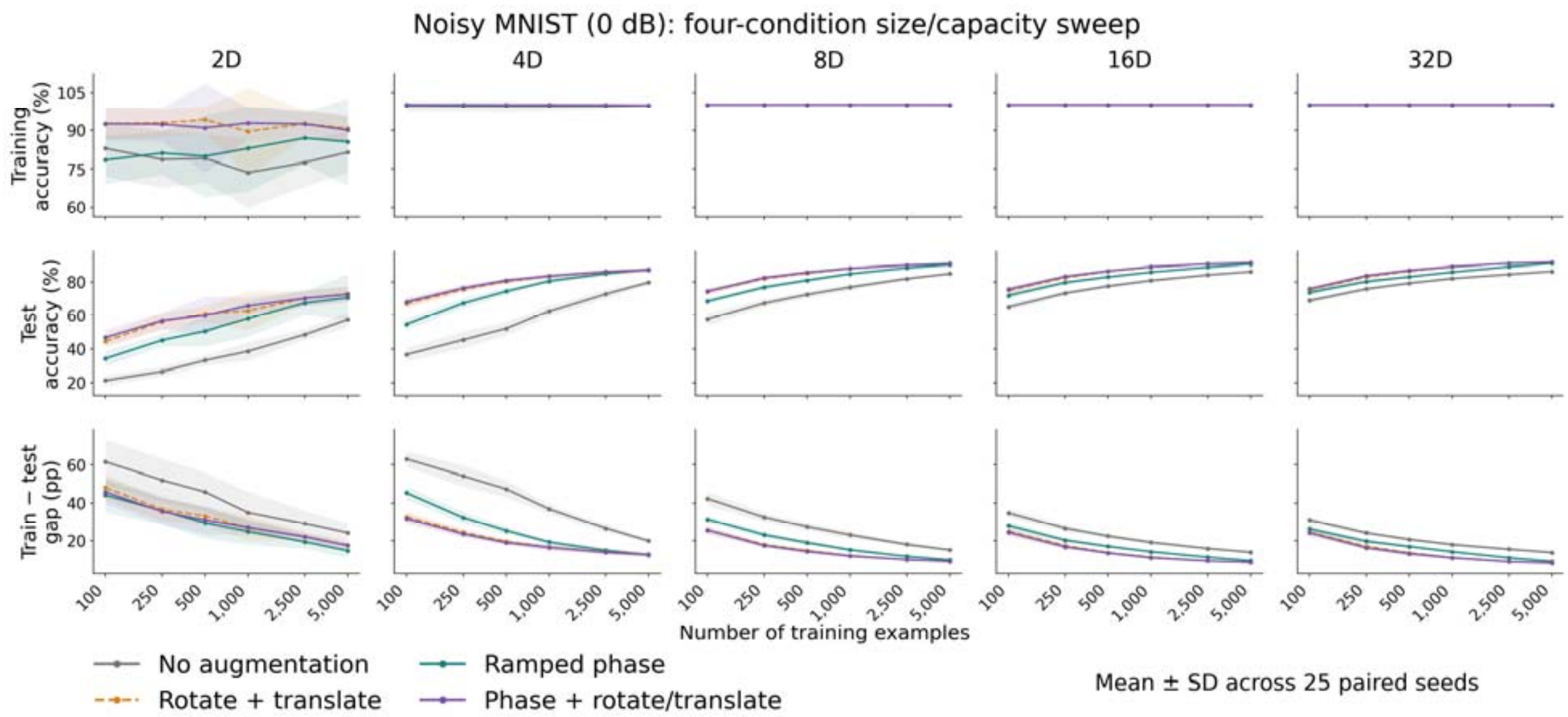

Figure S3. Phase and geometric augmentation in noisy MNIST across training-set sizes and latent dimensions. Columns show latent dimensions of 2–32, and rows show training accuracy, test accuracy and the train–test gap against training-set size on a logarithmic axis. Grey: no augmentation; teal: ramped phase augmentation; orange dashed: rotation and translation; purple: phase augmentation followed by rotation and translation. Lines and shading show mean ± 1 SD over 25 paired seeds. Accuracies are evaluated on unaugmented inputs, and test accuracy averages two noise views within each run.

accuracy by 10.3 percentage points over no augmentation, lower than the 14.3-point gain from rotation and translation. Adding phase augmentation to rotation and translation gave a modest further gain of 0.37 percentage points (averaged over settings; 95% interval across seeds 0.12–0.62). Mean test accuracy was higher in 24 of 30 settings, including 19 of the 20 settings with 1,000 or fewer training images, indicating a consistent benefit with limited training data.

Comparisons on UCI Synthetic Control. An additional study used a fixed stratified split (300 test traces, 60 validation traces and a 240-trace training pool), 60 labelled training traces per seed and a 60 → 32 → 16 → 6 classifier trained as in Table S1, with the ramp fixed between 1/28 and 0.25 cycles per sample. Over ten seeds (301–310), phase augmentation was compared with no augmentation, scalar amplitude scaling (factor drawn uniformly from 0.9–1.1), smooth time warping and four controls matched to the phase transform. Matched white noise has the expected total squared perturbation of the phase transform, and matched spectral noise has the same residual power at each frequency. Phase-moment Gaussian multipliers match the conditional mean and covariance of the complex Fourier multiplier, and the mean-phase filter applies the expected multiplier deterministically. Mean test accuracy was 93.07% without augmentation and 97.17% with phase augmentation, a gain of 4.10 percentage points (paired 95% interval 3.16–5.04). Time warping (97.03%) and the four matched controls (96.40–96.90%) gave similar gains, whereas scalar amplitude scaling (93.23%) did not.

bioRxiv preprint doi: https://doi.org/10.64898/2026.10.30.763149; this version posted November 1, 2026. The copyright holder for this preprint (which was not certified by peer review) is the author/funder, who has granted bioRxiv a license to display the preprint in perpetuity. It is made available under a CC-BY 4.0 International license.

Comparisons on noisy MNIST. An additional unsupervised study trained autoencoders (784 → 128 → 16 → 128 → 784) on 4,096 unlabelled noisy images for five seeds (301–305), by pixel mean-squared error with Adam (learning rate 0.001, batch size 256, 3,072 updates). Digits were low-pass tapered from 0.10 to 0.16 cycles per pixel, and Gaussian noise confined to 0.18–0.48 cycles per pixel was added at ten times the centred signal power (−10 dB). The input was an augmented noisy image and the target the unaugmented noisy image, with the ramp fixed between 0.16 and 0.28 cycles per pixel. The 16-dimensional representations of the 10,000 test digits, under two independent noise draws, were standardized, clustered by k-means into ten clusters and scored by the ARI against the digit labels. Phase augmentation raised mean ARI from 0.146 to 0.381 (paired 95% interval for the gain 0.217–0.253), higher than the 0.319 reached with rotation and translation and similar to matched spectral noise (0.379), phase-moment multipliers (0.382) and a known-cutoff low-pass filter followed by PCA (0.370). When the test noise was raised to −20 dB, spectral-noise augmentation gave a higher ARI than phase augmentation (0.281 versus 0.151). Intervals in both additional studies are paired t intervals across seeds, not adjusted for multiple comparisons.
